# Passive Virtual Reality During Post-Graft Burn Dressing Changes: A Randomized Controlled Trial

**DOI:** 10.1093/jbcr/irag033.578

**Published:** 2026-04-06

**Authors:** Alan D Rogers, Shahin Khodaei, Lilia Kaustov, Stephen Choi, Fahad Alam

**Affiliations:** Ross Tilley Burn Centre, Toronto, ON; Sunnybrook Health Sciences Centre, Toronto, ON; Sunnybrook Health Sciences Centre, Toronto, ON; Sunnybrook Health Sciences Centre, Toronto, ON; University of Toronto, Toronto, ON

## Abstract

**Background/Objective:**

Burn dressing changes remain among the most painful inpatient procedures and frequently require high-dose opioid analgesia. Although virtual reality (VR) distraction is recommended as an adjunct for procedural pain management, most prior studies focus on active VR and subjective pain outcomes. Evidence regarding passive VR and opioid-sparing effects remains limited. We evaluated whether passive immersive VR reduces opioid requirements during early post-autograft dressing changes.

**Methods:**

We conducted a prospective, randomized, open-label trial at a tertiary academic burn center. Adult patients with burn injuries undergoing autografting were randomized 1:1 to standard care or standard care plus passive VR using 360° immersive videos during two consecutive inpatient dressing changes. The primary outcome was cumulative intravenous morphine equivalents (IME) administered from 4 hours before to 4 hours after each dressing change. Secondary outcomes included pain and anxiety scores, sedation use, physiologic parameters, dressing efficiency, PTSD screening, 3-month opioid use, and patient and nurse satisfaction. Analyses were performed using an intention-to-treat approach with exploratory post hoc interaction analyses to assess the influence of baseline factors.

**Results:**

Eighty-two patients were randomized (41 VR, 41 control). Median cumulative intravenous morphine equivalents (IME) did not differ between groups (VR 45 mg [IQR 30–68] vs control 35 mg [20–55], *p*=.12; median difference 10 mg). There were no significant differences in opioid consumption before, during, or after individual dressing changes, nor in change in opioid requirements between the first and second dressing change. Pain and anxiety scores at all measured time points were similar between groups, as were rates of conscious sedation, physiologic parameters during procedures, PTSD screening outcomes, and opioid use at 3 months.

Despite the absence of opioid sparing, patient-centered outcomes were favorable: over 90% of participants reported enjoying the VR experience and would recommend its use, with perceived anxiety reduction reported more frequently than pain relief. Nursing staff reported high usability and minimal interference with dressing change workflow.

**Conclusions:**

Passive VR did not reduce opioid requirements or procedural pain during early post-graft dressing changes. However, high patient and staff satisfaction suggests perceived benefit and feasibility for clinical implementation in selected contexts.

**Applicability of Research to Practice:**

Passive VR is feasible and scalable but should not be expected to replace pharmacologic analgesia for highly painful inpatient procedures. It may be better suited to lower-pain interventions such as therapy sessions, mobilization, or outpatient wound care.

**Funding for the study:**

This study was supported by the organisation’s Innovation Fund. Funding facilitated study coordination and acquisition of virtual reality equipment. The funding body had no role in study design, data collection, data analysis, interpretation of results, or decision to submit for publication.

